# Occurrence and Analysis of Thermophilic Poly(butylene adipate-co-terephthalate)-Degrading Microorganisms in Temperate Zone Soils

**DOI:** 10.3390/ijms21217857

**Published:** 2020-10-23

**Authors:** Jana Šerá, Markéta Kadlečková, Ahmad Fayyazbakhsh, Veronika Kučabová, Marek Koutný

**Affiliations:** 1 Department of Environmental Protection Engineering, Faculty of Technology, Tomas Bata University in Zlín, T. G. Masaryka Square 5555, 760 01 Zlín, Czech Republic; sera@utb.cz (J.Š.); kucabova@utb.cz (V.K.); mkoutny@utb.cz (M.K.); 2 Department of Physics and Materials Engineering, Faculty of Technology, Tomas Bata University in Zlín, T. G. Masaryka Square 5555, 760 01 Zlín, Czech Republic; m1_kadleckova@utb.cz

**Keywords:** poly(butylene adipate-co-terephthalate), soil, compost, biodegradation, actinomycetes

## Abstract

The ubiquity and character of thermophilic poly(butylene adipate-co-terephthalate) (PBAT)-degrading microorganisms in soils were investigated and compared to the process in an industrial composting plant. PBAT degraders were sought in 41 temperate zone soils. No mesophilic degraders were found by the employed method, but roughly 10^2^ colony-forming units (CFUs) of thermophilic degraders per gram of soil were found in nine soils, and after an enrichment procedure, the PBAT-degrading consortia were isolated from 30 out of 41 soils. Thermophilic actinomycetes, *Thermobispora bispora* in particular, together with bacilli proved to be the key constituents of the isolated and characterized PBAT-degrading consortia, with bacilli comprising from about 30% to over 90% of the retrieved sequences. It was also shown that only consortia containing both constituents were able to decompose PBAT. For comparison, a PBAT film together with two types of PBAT/starch films were subjected to biodegradation in compost and the degrading microorganisms were analyzed. Bacilli and actinobacteria were again the most common species identified on pure PBAT film, especially at the beginning of biodegradation. Later, the composition of the consortia on all three tested materials became very similar and more diverse. Since waste containing PBAT-based materials is often intended to end up in composting plants, this study increases our confidence that thermophilic PBAT degraders are rather broadly present in the environment and the degradation of the material during the composting process should not be limited by the absence of specific microorganisms.

## 1. Introduction

**Figure 1:**
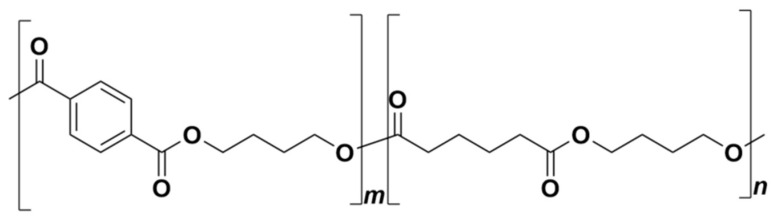
Poly(butylene adipate-co-terephthalate) (PBAT) chemical structure.

Nowadays, plastic waste constitutes a severe issue reflected by both experts and the public. Biodegradable polymers represent an alternative with the potential to replace conventional polymers in several specific applications [1,2]. Today, they are increasingly being used, especially in the agricultural sector [3,4,5,6,7]. Poly(butylene adipate-co-terephthalate) (PBAT) is one of the biodegradable polymers already commercialized under different trading names (Figure 1) intended specifically for applications in agriculture and packaging. It can be roughly characterized as a synthetic fossil resources-based aromatic–aliphatic co-polyester with semi-crystalline morphology [8,9]. It is manufactured by the polycondensation of 1,4-butanediol and the mixture of adipic and terephthalic acids. PBAT has been presented as biodegradable material that leaves no toxic metabolites behind and causes no harmful effects to the environment [10,11]. The first step in PBAT degradation is believed to be enzymatic hydrolysis by microbial extracellular enzymes, which cleave the ester bonds and release oligomers and monomers to be further metabolized inside the microbial cells [12,13]. This may be preceded or accompanied by physical and chemical processes such as abiotic hydrolysis or photodegradation [14,15,16]. The identified degrading strains belong mostly to the class *Actinobacteria*, e.g., *Thermobifida fusca* [17], *Thermomonospora fusca* [18], *Thermobifida cellulosilytica* [19], *Saccharomonospora viridis* [20], and *Thermobifida alba* [21]. It seems evident that microbial consortia can achieve complete degradation of PBAT under industrial composting [18,22]. A thermophilic actinobacterium, *Thermobifida fusca*, was isolated and identified as a PBAT degrader under composting conditions [17]. Morro et al. [13] studied the biodegradation and photodegradation of a PBAT/poly(lactic acid) (PLA) blend under thermophilic conditions. Their results showed that the two main factors that have the potential to enhance bacterial biodegradation were photodegradation and the use of the appropriate bacterial strains. Their results led them to conclude that biodegradation was faster when *Bacillus subtilis* was present.

The degradation of pure PBAT by mesophilic bacteria has been the topic of a few papers; however, only partial degradation of the material was observed even after a prolonged incubation [23,24,25]. In this case, some mesophilic and thermophilic microorganisms showed their potential to degrade PBAT [24]. Bonmee and his colleagues [26] studied the biodegradation rate of four different polymers: PBAT, poly (3-hydroxybutyrate-co- 3-hydroxyvalerate) (PHBV), PLA, and poly(butylene succinate) (PBS) under anaerobic and oxygen-limited conditions. Their results showed that the PBAT biodegradation rate was the lowest among all. Previous research has shown that this rate under composting conditions is high, e.g., in one study, they found 95% degradation in 124 days [27].

Fungi degraders of the material were also identified, namely *Humicola insolens* and *Isaria fumosorosea* [19,25], and enzymes able to depolymerize PBAT were found in bacteria isolated from freshwater and from wastewater treatment sludge [28,29]. Saadi et al. [30] studied the fungal degradation of PBAT in both compost and soil. They reported that the biodegradation rate is more related to the temperature of the medium than the use of compost. This result led them to conclude that the PBAT structure needs to be hydrolyzed before fungi can proceed with the biodegradation.

It seems that both thermophilic and mesophilic bacterial enzymes preferentially catalyze the cleavage of the bonds between aliphatic components of the co-polyester [13]. The hydrolysis of ester bonds between butanediol and terephthalate units proceeds much slower than the hydrolysis of ester bonds between adipate and butanediol [10,23,31], so the proportion of the aromatic component can directly influence the rate of biodegradation. Sera et al. [32] studied the influence of starch blended with PBAT on PBAT biodegradation in soil under mesophilic conditions. Their results revealed that preferential starch degradation enhanced the active surface area of the polymer, which supported PBAT biodegradation. Also, the starch content could provide some extra nutrients for the PBAT degrading consortium; hence, the process might exhibit some form of co-metabolism. Seligra et al. [33] studied the influence of starch nanoparticles blended into PBAT/TPS (TPS, thermoplastic starch) films on their biodegradation. They observed that the addition of nanoparticles enhanced the moisture content of the films and water diffusion into the polymer, and consequently supported the biodegradation by increasing the potential for microbial attack. Muroi et al. [11] investigated the microbial degradation of PBAT over seven months in soil. After that time, they identified *Azospirillum* and *Mesorhizobium* as the two main bacterial PBAT degraders.

In the present study, we investigated the presence of PBAT degraders in soils and compared the findings with the composition of the microbial community during the biodegradation process, as observed in compost obtained from an industrial composting plant. A series of methods were used, from classical microbiology methods (liquid and solid media cultivation and isolation, microscopy) to molecular biology techniques. The majority of the products currently present on the market that are made from PBAT are expected to end up in industrial composting processes. Soil represents a natural source and reservoir of microorganisms with the potential to degrade PBAT. The main purpose of this study is to investigate whether such strains are broadly present or rather scarcely distributed in the environment, which is both important and interesting. The subsequent goal is an attempt to isolate, identify, and characterize such strains or degrading consortia.

## 2. Results

### 2.1. Screening for PBAT Degraders in Soils

The soil types included in this study comprised the most common ones found in the eastern part of the Czech Republic, namely fluvisols, cambisols, and luvisols. Some basic parameters of the sampled soils are summarized in Table 1. pH varied in the interval from 5 to 7, with the majority of values close to 6. Organic carbon values found in the soil samples indicate an equilibrium between organic matter income and decomposition [34].

Microorganisms, i.e., bacteria and fungi, capable of PBAT degradation present in the studied soils were detected on plates with the polymer as the sole carbon source by observing the clear zones of depolymerized PBAT surrounding the active colonies in the otherwise opaque medium. The mechanisms of this technique are such that the microorganisms in the colony produce water-soluble extracellular enzymes, which diffuse into the colony surroundings and catalyze the cleavage of the ester bonds in the polymer. As a consequence, the polymer microparticles suspended in the medium dissolve and the medium turns from opaque to transparent. It must be stressed that the formation of clear zones is directly connected to the hydrolysis of polymer macromolecules; thus, the detection method is not based on the simple growth of colonies but comprises a specific proof of polymer degradation [15]. Also, the diameter of the clear zone can be used as a semi-quantitative measure of the depolymerase activity of the colony. The detection limit of the method was about 100 colony-forming units (CFU) per gram of dry soil weight based on the sample preparation procedure and the volume of the suspension spread on plates. Firstly, we attempted several times, and with some modifications of the isolation media, to detect mesophilic PBAT-degrading microorganisms at 25 °C as colonies forming clear zones on PBAT-containing media as described, but the results were negative in all investigated soils. Also, after performing the enrichment procedure described below, no mesophilic PBAT-degrading microorganisms were found in any of the soils. It was hence concluded that mesophilic PBAT-degrading microorganisms are either not present or their concentrations are too low to detect in the investigated soils.

The presence of thermophilic degraders at 58 °C was confirmed in 9 soils out of 41; however, the counts were just above the detection limit. The actual numbers of the degraders thus corresponded to the lower hundreds of CFU per gram of soil. From the known basic soil properties, the presence of the degraders tended to correlate with increasing soil pH (*p* = 0.02).

In order to investigate whether the thermophilic PBAT degraders were present in other soils at lower levels, the soil samples were incubated at 58 °C in the presence of a PBAT microparticle suspension, which was prepared using an identical procedure for the PBAT agar media, with the aim of supporting the growth and enrichment of the thermophilic PBAT-degrading microorganisms. After the incubation, the soil suspensions were plated on PBAT-amended mineral agar plates. After the enrichment procedure, the degraders were found in 30 out of 41 soils in counts ranging from 10^2^ to 10^4^ CFU per gram of soil, and now had no clear correlation either with pH or CFU counts before the enrichment. The above-described results provided strong evidence that thermophilic PBAT-degrading microorganisms are rather ubiquitous in soils and do not represent a particular limitation for the treatment of PBAT-containing waste materials in composting processes.

### 2.2. Characterization of the PBAT-Degrading Consortia

The next step of the study was to isolate and characterize the thermophilic degrading microorganisms or consortia. This proved to be much more difficult than anticipated. After a microscopic inspection, it was concluded that the clear zone colonies contained filamentous bacteria with morphology strongly suggesting that they could be actinomycetes, with the significant presence of other rod-shaped bacteria later identified as various thermophilic bacilli.

Optical microscopy of the consortia also suggested the absence of fungi, which is not so surprising considering the relatively high incubation temperature (58 °C), which is not tolerated by most fungi. After several failed attempts to isolate pure actinomycetes on media with PBAT alone or in combination with various growth factors, their mixtures, malt extract as a co-substrate, or on the specialized commercially-available media for the isolation of actinomycetes, we had to conclude that these isolation attempts resulted either in the elimination of actinomycetes from the microbial consortia and also the absence of the PBAT degradation capacity of the consortium, or the actinomycetes were still contaminated with bacilli, but the resulting consortium was able to degrade the polymer. It was thus deduced that an actinomycete is a necessary component of the degrading consortia, as also suggested by Muroi et al. [11], however it is not able to grow on the polymer as a pure strain but only as a member of a consortium. This is consistent with the finding that actinomycetes isolated during PBAT degradation in compost were not able to utilize respective monomers and oligomers and were more effective as members of the degrading consortia [18]. Subsequently, it was decided to isolate degrading consortia by transferring the biomass from the center of individual clear zones, spreading it onto new PBAT plates, and repeating this procedure several times while checking for polymer degradation activity. The resulting purified PBAT-degrading consortia were further characterized.

Firstly, PCR-DGGE (Polymerase chain reaction—Denaturing gradient gel electrophoresis) was used to analyze the composition of the PBAT-degrading consortia isolated from individual soil samples. This method is known to overrepresent the major components of the consortia [35], which could be an advantage in this case.

**Figure 2:**
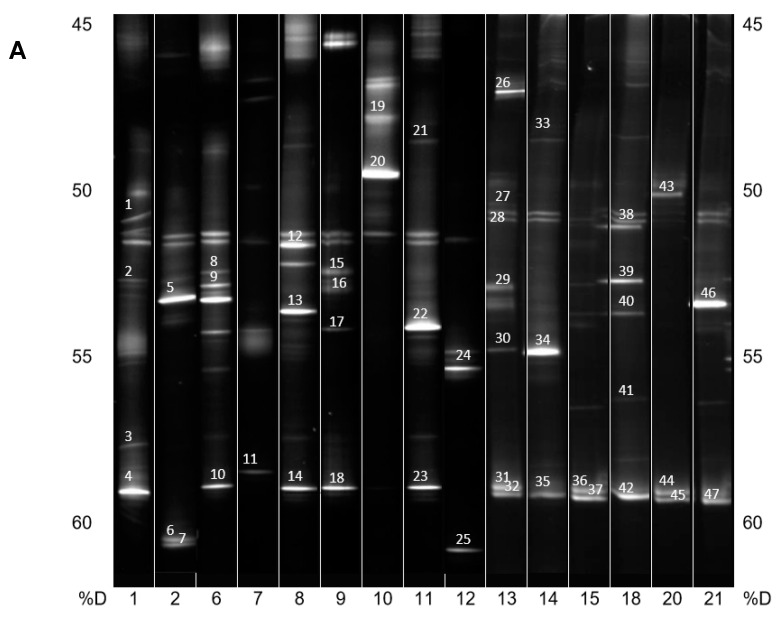

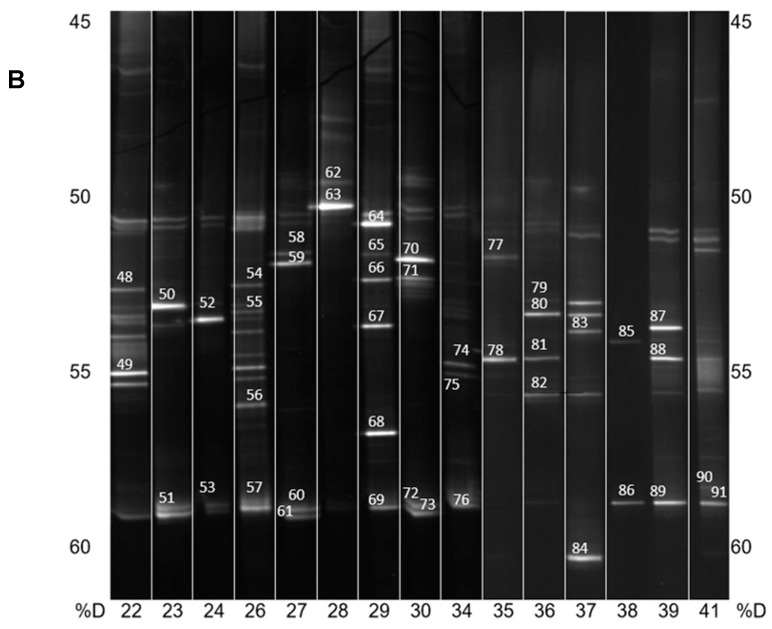
(**A**) PCR-DGGE analysis of isolated PBAT-degrading consortia. The numbers on the bottom represent the soil samples from Table 1. Identified bands are numbered, and their taxonomical assignation can also be found in Table 1. %D: the percentage of denaturation agent in the gradient gel; (**B**) Continuation of (**A**).

DNA from the consortia were isolated and V3 to V5 fragments of the 16S rRNA gene were PCR amplified and resolved by DGGE (Figure 2A,B). The most intensive bands were sequenced, and their taxonomy was allocated (Table 1).

**Figure 3:**
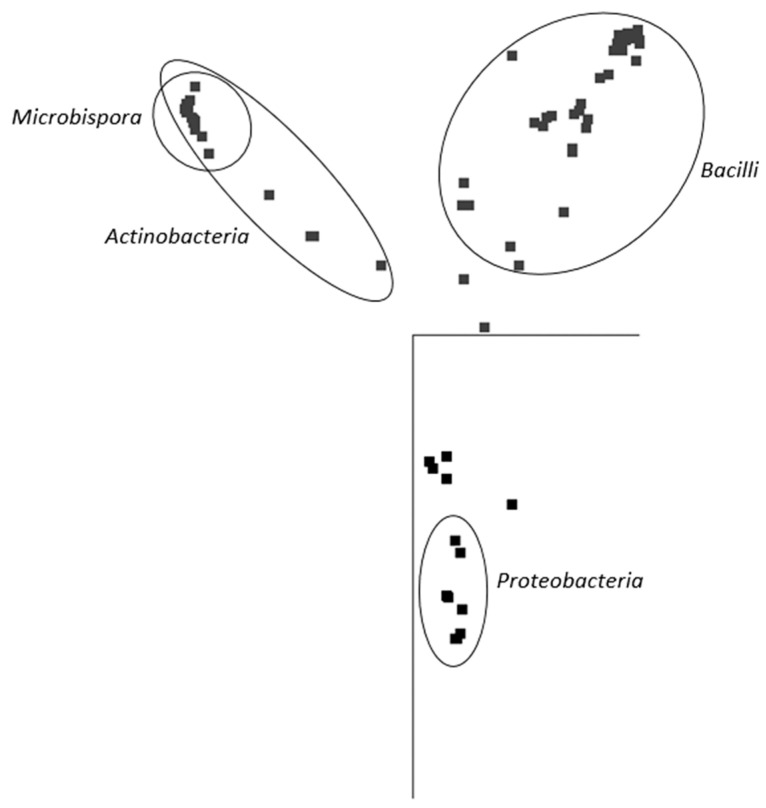
Principal component analysis (PCA) based on sequences retrieved from DGGE gels. The representation of the data in two-dimensional space with the use of two main components of variability is visualized. Points represent individual sequences of distinct bacterial species retrieved from the DGGE gel (Figure 2); similar sequences are close to each other. Ellipses cluster sequences belonging to the inscribed taxonomical groups. JalView 2.10 freeware was used to analyze the sequences [36].

In general, and with some simplification, a band in the gel represents a bacterial species, and its intensity is roughly proportional to its abundance in the consortium. It is evident that in the majority of PBAT-degrading consortia, at least one signal could be assigned to some actinomycete strain (most often *Thermobispora bispora*). Signals from this species tended to show at the bottom of the gel because of the very high GC content of their DNA, which is typical for actinobacteria. In some other consortia (consortia 22 and 36), such signals were also clearly visible but were not successfully sequenced and hence could not be positively identified. Therefore, *Thermobispora bispora* appears to be a prominent thermophilic PBAT degrader broadly distributed in soils, at least in the studied area. Among the signals other than those of *Thermobispora bispora*, bands belonging to various thermophilic bacilli were dominant in the consortia, with *Geobacillus* as the most frequent genera. This is not entirely surprising because this group is generally highly present in soils and members of the group are often thermotolerant or thermophilic. Other bacterial groups were scarcely distributed and identified among the isolated consortia, namely proteobacteria. By evaluating the random nature of the proteobacteria members present, it was suggested that these bacteria are either present accidentally or opportunistically to exploit polymer degradation products left by actinobacteria and bacilli. With the help of principal component analysis (PCA), the retrieved sequences could be clustered into three general groups, as seen in Figure 3. It is evident that actinomycetes, and particularly *Thermobispora bispora* and various bacilli, represented the most distinct clusters and comprised the majority of the identified microorganisms.

**Figure 4:**
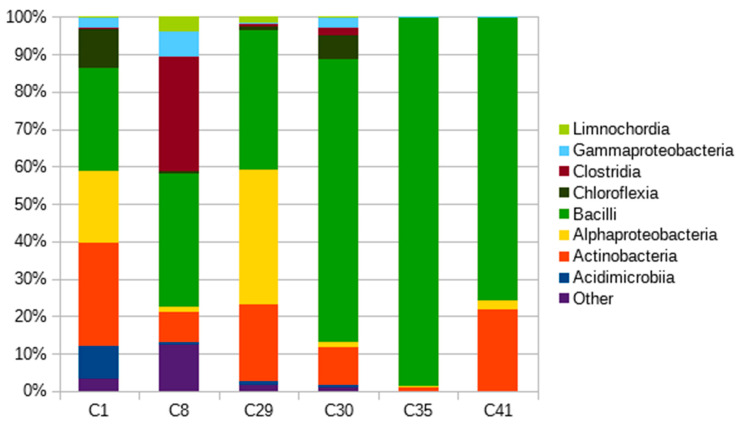
Taxonomical compositions of PBAT-degrading consortia on a Class taxonomic level.

Six selected polymer-degrading microbial consortia isolated from soils belonging to three of the most common soil types included in the study (luvisol, fluvisol, and chernozem) and four habitats (field, stream bank, orchard, and grass cover) [37] were also analyzed using next generation sequencing (NGS). NGS helped to obtain quantitative data describing the composition of the microbial consortia at a higher taxonomical level (Class, Figure 4). Bacilli were the prevailing class, followed by actinobacteria and alphaproteobacteria. Bacilli accounted for the majority of identified sequences in all consortia, but were especially dominant in the consortia derived from field soils under intensive agricultural management (99% of bacilli in consortium C35), reflecting a possibly lower diversity of the microbial community in these soils [38]. Consortia C1, C8, and C29 reflected much higher diversity, but bacilli and actinobacteria are strongly present. It can also be noted that these consortia originated from the sites with more diverse plant cover. Actinobacteria were detected in all consortia, with prevalence ranging from 8% to 27%. However, in consortia C35, only 1% of actinobacteria were present, and all other bacterial taxonomical classes were well under 1%. These findings support our theory about the importance of actinobacteria in PBAT biodegradation.

### 2.3. Characterization of the PBAT-Degrading Community from Compost

Equipped with some knowledge about the composition of the potential thermophilic degrading consortia that can arise from soils, we decided to investigate PBAT biodegradation in a mature compost environment sampled from a local industrial composting plant.

**Figure 5:**
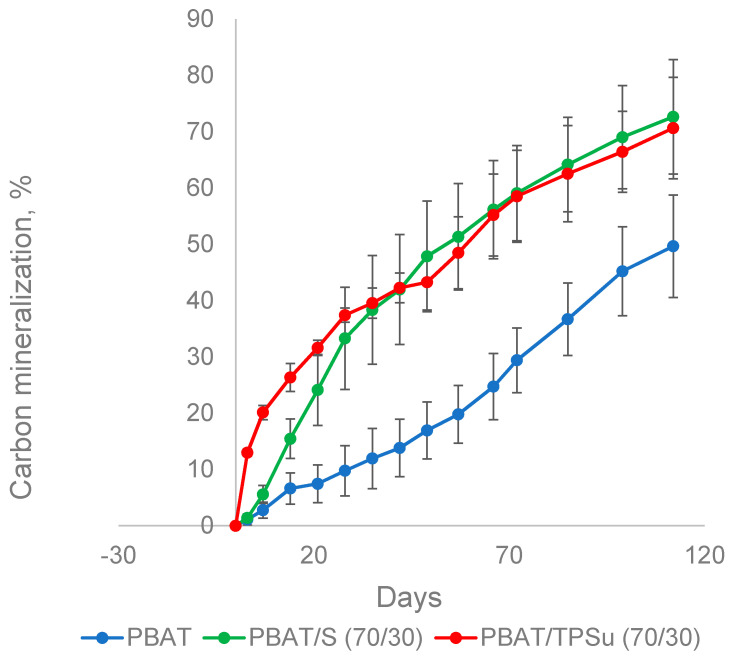
Biodegradation of PBAT-based materials in a composting environment at 58 °C. S, non-plasticized starch; TPSu, thermoplastic starch with urea as plasticizer.

In addition to the film made of PBAT alone, blended films containing 30% powder or thermoplastic starch were also investigated, representing the most commercially available types of these materials. The materials were incubated in the presence of the mature compost at 58 °C for about 100 days, and their biodegradation was monitored by the measurement of CO_2_ (Figure 5). PBAT/TPSu urea (TPSu, thermoplastic starch with urea as plasticizer) degradation was fastest during the first 35 days of incubation, without or with a very short lag phase. This was probably caused by the fast mineralization of the low molecular weight plasticizers and a more loose structure of the thermoplasticized starch [39]. The PBAT/starch blend was mineralized faster than pure PBAT in the beginning, reflecting the easy decomposition of the starch component. However, from about day 40, the slopes of all curves were quasi identical. Both blends reached around 70% mineralization at the end of the experiment (112 days); PBAT film mineralization was about 50%. It seems that the TPS and starch in the blend with PBAT did not accelerate pure PBAT biodegradation under simulated industrial composting conditions; microorganisms probably just preferably consumed the easily degradable components first. PBAT was decomposed only when starch or TPS were no longer available in the blends.

**Figure 6:**
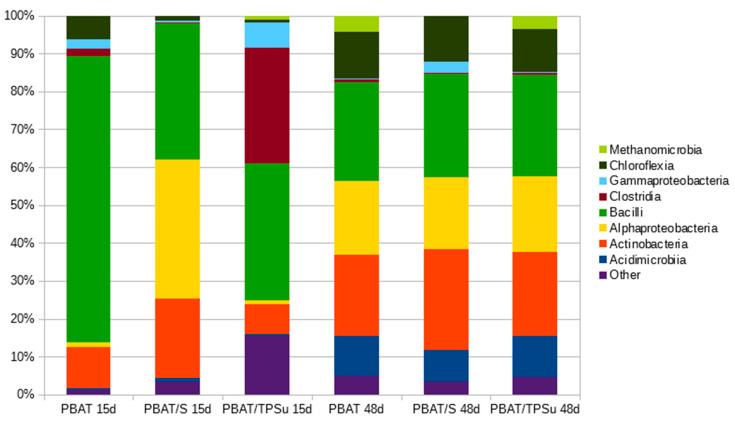
Taxonomical compositions of biofilms from the surface of the PBAT based materials at Class taxonomic level during their biodegradation in compost at 58 °C. The materials were sampled after 15 days (15d) and 48 days (48d) of biodegradation.

Samples of the materials were withdrawn from the compost incubation at days 15 and 48, gently rinsed, and subjected to DNA isolation and sequencing (Figure 6). The aim was to obtain information about the composition of the bacterial biofilms covering the materials and compare them with the data obtained for the consortia that were analyzed previously. For such a comparison, the most interesting data are from day 15 of the pure PBAT material, because the consortium should reflect the initial stage of the polymer degradation. In this case, the bacterial community was dominated by bacilli with the important presence of actinobacteria, which is in accordance with the conclusion drawn from the consortia analyses (that the combination of these two classes was important for PBAT biodegradation, at least at the beginning of the process). Later (PBAT day 48), other bacterial groups stepped in, namely alphaproteobacteria, acidimicrobia, and chloroflexi, taking advantage of the now partially-degraded polymer and its degradation products. For the materials containing starch, an easily degradable substrate by many bacterial groups, the consortia present were also more diverse in the beginning. An interesting feature was the remarkable similarity of the bacterial consortia at day 48 for all investigated materials. At this time, the starch component and the plasticizer were already consumed, and so the consortium should reflect the later stage of polymer degradation, with both suggested primary polymer degraders (actinomycetes and bacilli) and the secondary degradation product consumers (e.g., alphaproteobacteria).

**Figure 7:**
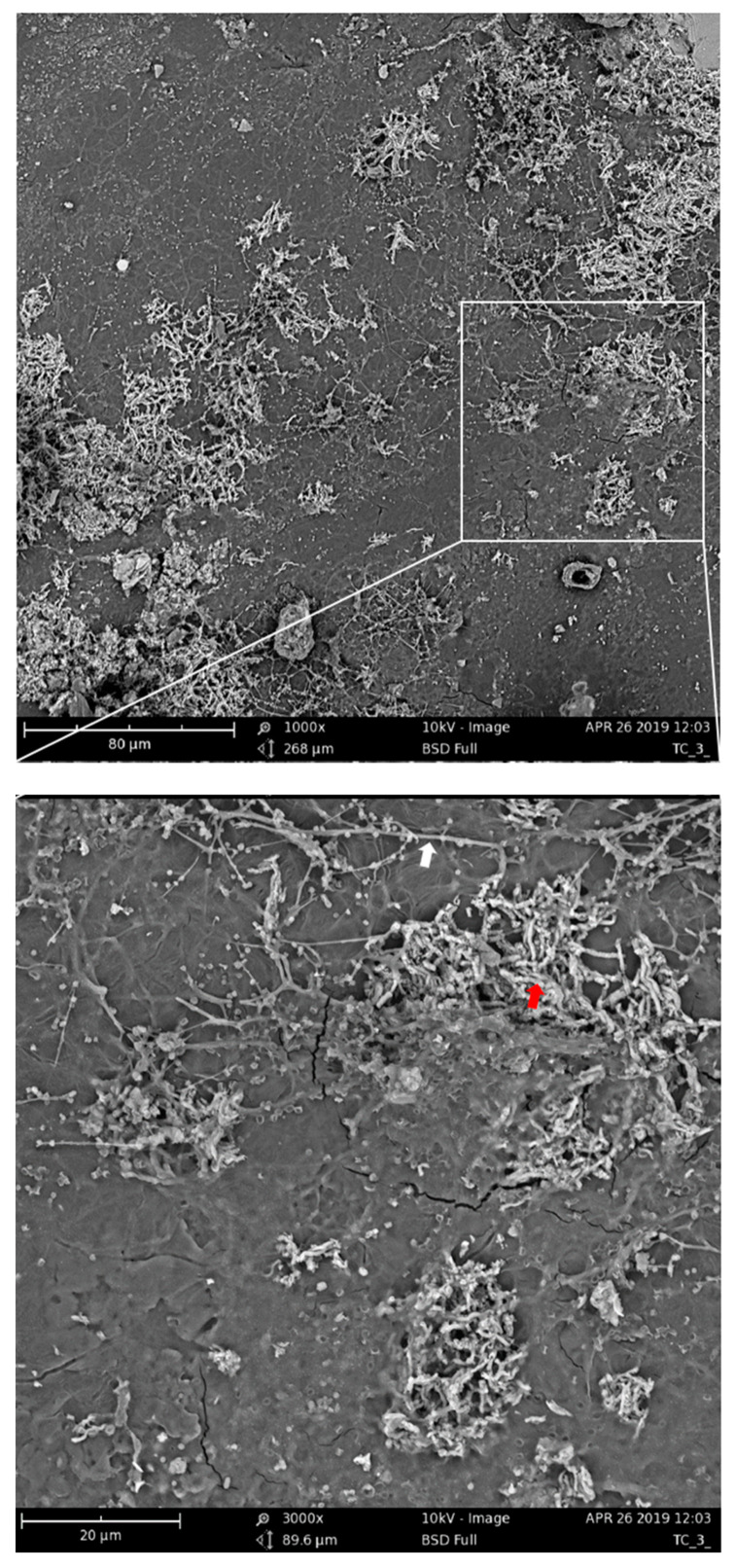
Scanning electron microscopy of PBAT films incubated for nine days in compost at 58 °C. The white arrow indicates putative actinomycete filaments; the red arrow indicates putative bacilli cells.

The assumption of the importance of actinomycetes and bacilli for the PBAT biodegradation process was further supported by the microscopic observation of the PBAT film surface during the incubation in compost. After nine days of incubation of the material in compost, filamentous bacteria with spores that could be morphologically identified as actinomycetes were spread on the surface (Figure 7, white arrow). Bacilli cells were less morphologically distinguishable, but their morphology was consistent with rather thick connected rods forming dense microcolonies (Figure 7, red arrow).

## 3. Materials and Methods

### 3.1. Polymer Materials

PBAT from Jinhui Zhaolong, China was used in the study. Samples of polymer films (100 µm) used for compost incubation were prepared in Polymer institute of Slovak academy of sciences (Bratislava, Slovakia) by extrusion and compression molding as earlier described [40]. Neat PBAT, PBAT (70%) with powder starch (30%) (PBAT/S), and PBAT (70%) with thermoplastic starch (TPS) (30%) and urea (0.6%) as a plasticizer (PBAT/TPSu) was used in the study.

### 3.2. Soil and Compost Characterization

The soil sampling locations were mostly selected from the eastern part of the Czech Republic from spots under regular agricultural management, where data about the soil type and quality are available. Samples of 41 soils were collected, mostly fluvisols, cambisols, and luvisols, as they were the most frequent soil types in the area. About 20 cm was sampled from the surface layer; approximately 5 cm of the thick surface layer rich in plant material was discarded. Mature compost samples were obtained from the municipal composting facility (Suchý důl, Zlín, Czech Republic). The pH and dry weight of the materials were determined according to ISO 10390:2005, and ISO 11465:1993, respectively.

### 3.3. PBAT Agar Plates and Enrichment of Thermophilic PBAT Degraders

PBAT (2 g) was dissolved in 20 mL of chloroform (Merck, Kenilworth, NJ, United States). The PBAT solution was poured into 80 mL of distilled water containing 0.5% (*w*/*w*) of PVA (Merck, Kenilworth, NJ, United States) and blended in a homogenizer (24,000 RPM, 5 min) and then sonicated (2 min). The emulsified solution was then incubated at 25 °C overnight under gentle continuous stirring to remove the organic solvent [41]. The resulting aqueous suspension contained solid PBAT microparticles (1–10 µm, optical microscopy direct observation). The particles were collected by centrifugation (12,000 g, 10 min), washed twice with demi water and finally suspended in 50 mL of demi water to a concentration of about 40 mg of PBAT in 50 mL.

The composition of the mineral medium was as follows: 3.8 g Na_2_HPO_4_·12H_2_O, 1.8 g KH_2_PO_4_, 0.02 g MgSO_4_·7H_2_O, 0.03 g Fe(NH_4_)_2_(SO_4_)_2_·6H_2_O, 0.01 g CaCl_2_·2H_2_O, 0.5 g NaCl, 0.3 g NH_4_Cl and 1 mL of trace element solution in 1 L. The trace element solution contained 0.20 g MnSO_4_, 0.029 g, H_3_BO_3_, 0.022 g ZnSO_4_·7H2O, 1.0 g Na_2_MoO_4_, traces of Co(NO_3_)_2_, and traces of CuSO_4_ dissolved in 500 mL of water (all chemicals by Merck, Kenilworth, NJ, United States). To prepare solid media with the polymer solution, 100 mL of mineral medium, 20 mL of PBAT suspension, 4 mg of yeast extract (HiMedia, Mumbai, India), and 2.2 g of agar (HiMedia, Mumbai, India) were used.

Degraders of PBAT were selectively enriched at composting temperature (58 °C) in a mixture of soil (15 g of dry weight), perlite (2 g), and a co-polyester microparticle suspension (2 mL) at 50% relative humidity in 500 mL biometric flasks for 64 days. After 64 days, microorganisms from this mixture were suspended and incubated on agar plates with PBAT as a sole carbon source at 58 °C for ten days. Correlation analysis and the calculation of Pearson coefficients and *p* values were done with the open source ggpubr R package (by Alboukadel Kassambara).

### 3.4. Biodegradation Test in Compost

The method used was based on previously published protocols with some modifications [32]. Polymer films (100 mg), perlite (2.5 g), and compost (1.75 g dry weight) were weighed into 500 mL biometric flasks. The flasks were sealed with stoppers equipped with septa and incubated at 58 °C. Separate incubations were prepared for scanning electron microscopy (SEM) and molecular biology analysis. The measurements of carbon dioxide (CO_2_) production were made weekly by using a UGA 300 High-Pressure Gas Analyzer (Stanford Research Systems, Sunnyvale, CA, USA) equipped with an electron multiplier detector. 

Concentrations of CO_2_ were derived from the calibration curve, and the percentage of mineralization pertaining to the initial carbon content of the sample was calculated [32]. The carbon content of the polymer samples was determined by a Flash Elemental Analyzer 1112 (ThermoFisher Scientific, Waltham, MA, USA). Three parallel flasks were run for each sample, along with four blanks.

### 3.5. Scanning Electron Microscopy

Scanning electron microscopy (Phenom Pro Desktop SEM, ThermoFisher Scientific, Waltham, MA, USA) was carried out to observe surface changes in the degraded films. All samples were coated with a thin layer of Au/Pd. The microscope was operated in high vacuum mode at an acceleration voltage of 10 kV.

### 3.6. DNA Analysis

DNA was isolated using the DNeasy PowerSoil DNA extraction kit (Qiagen, Hilden, Germany) and amplified by nested PCR. In the first PCR round, universal bacterial primers fD1 and rD1 (AGAGT TTGAT CCTGG CTCAG, and AAGGA GGTGA TCCAG CC, respectively) were used to amplify nearly the full length of the 16S rRNA gene [42]. Subsequently, 1 μL of the first PCR round was used as a template for nested PCR amplification of the V3–V5 hypervariable region of the 16S rRNA gene with the primer pair 341fGC and 907r (ATTAC CGCGG CTGCT GG, and CCGTCAATTCCTTTGAGTTT, respectively), where the GC clamp (CGCCC GCCGC GCGCG GCGGG CGGGG CGGGG GCACG GGGGG) was covalently attached to the 5′ end of the forward primer [43,44]. PCR cycles were designed according to Husarova et al. [44]. After each PCR amplification round, the size of the PCR product was verified on a 1% agarose gel.

Denaturing gradient gel electrophoresis (DGGE) is a molecular technique for the rapid analysis of microbial community diversity, composition, and dynamics. This technique also has the potential to analyze and compare multiple samples simultaneously. DGGE separation of the amplified PCR products was achieved using a Cipher DGGE system (C.B.S. Scientific, San Diego, CA, USA). In principle, the method is able to separate DNA fragments of identical length according to differences in their nucleotide sequences. With some simplification, individual bands in the gel thus correspond to individual distinct bacterial species. Radical polymerization of the gel was achieved by the addition of tetramethyl ethylene diamine (TEMED) and ammonium persulfate (APS). PCR products from the second amplification process, together with 100-bp DNA ladder (NEB, Ipswich, MA, USA) which served as a marker [45], were loaded onto the denaturing gel, with an optimal gradient of 40–80% of DNA denaturing compounds. Urea and formamide were used as denaturing reagents. Electrophoretic separation of the DNA fragments was performed at a constant voltage of 90 V for 16 h. After the separation procedure, the electrophoresis gels were stained with GelRed (Biotium, Hayward, CA, USA) according to the manufacturer’s instructions and documented. The whole procedure was repeated twice with identical results. Relevant bands were excised, DNA eluted, and re-amplified with the same primers, but without the GC clamp, and sequenced. Selected sequences were subjected to principal component analysis with the help of JalView software [36].

DNA isolated from selected samples of enriched soils and from simulated industrial composting incubations were analyzed by next generation sequencing (NGS). The V4–V5 hypervariable region of the 16S rRNA gene with the bacterial primer pair V4–V5 515F and V4–V5 R926 (GTGYCAGCMGCCGCGGTAA and CCGYCAATTYMTTTRAGTTT, respectively, with a universal overhang) was sequenced. Product sizes and concentrations were verified by agarose electrophoresis. The library was sequenced on a MiSeq (Illumina, San Diego, CA, USA) using the v2 version of chemistry, and 250 nt paired-end read settings. The data were further processed with SEED v2.1.05 [46] and the Phyloseq R package [47], and the taxonomy was assigned using the SILVA reference database [48].

## 4. Conclusions

PBAT degraders were sought in 41 temperate zone soils. The isolated consortia were further analyzed with microbiological and molecular biology methods, including sequencing, and compared with the data obtained during the biodegradation of PBAT-based materials under industrial composting conditions. The concluding remarks are as follows:

1. Mesophilic PBAT degraders are not present, or were not detected by the employed method due to their absence or extremely low numbers. On the contrary, the thermophilic degraders, although present in relatively low numbers in the investigated soils, are broadly present in the environment and so the availability of specific PBAT degraders does not represent a limiting factor for the composting treatment of this synthetic polymer.
2. Thermophilic actinomycetes, *Thermobispora bispora* particularly, together with bacilli proved to be the key constituents of the isolated and characterized PBAT-degrading consortia as derived from both PCR-DGGE and NGS analysis of the isolated consortia. Similar patterns were also observed in the PBAT-degrading microbial community from the local composting plant.
3. The attempts to isolate pure strains with the polymer as the sole carbon source failed, which suggested that the process requires the participation of more microorganisms.
4. Actinomycetes and bacilli were the prevalent species in the initial phase of biodegradation in compost. Moreover, from microscopic analysis, filamentous bacteria with spores that could be morphologically assigned as actinomycetes were observed after nine days of incubation. These microscopy results, in conjunction with other evidence including experiments on agar plates, DGGE analysis and identification of the retrieved sequences, and NGS results, lead us to conclude that actinomycetes are important for PBAT biodegradation in compost.

## Figures and Tables

**Table 1:** Basic parameters of sampled soils and identification of microorganisms.

Soil No.	Habitat	^a^ C_org,_ %	Soil Type	pH	^b^ CFU 1 × 10^2^ Per Gram	^c^ CFU 2 × 10^2^ Per Gram	Band Number	Identified Bacteria	^d^ %
1	Orchard	1.3	Fluvisol	6.60	2	44	1.1	*Anoxybacilluss* sp.	97
							1.2	*Paenibacillus*	92
							1.3	*Thermobispora bispora*	97
							1.4	*Thermobispora bispora*	97
2	Stream bank	1.3	Fluvisol	5.85	<1	9.9	2.5	*Geobacillus* sp.	99
							2.6	*Thermobispora bispora*	97
							2.7	*Thermobispora bispora*	97
3	Field	2.3	Luvisol	6.2	<1	<1	-	*-*	-
4	Field	3.0	Fluvisol	6.41	<1	<1	-	*-*	-
5	Field	3.0	Fluvisol	6.59	<1	<1	-	*-*	-
6	Grass cover	0.8	Luvisol	6.10	<1	5.7	6.8	*Aeribacillus* sp.	96
							6.9	*Geobacillus caldoxylosilyticus*	99
							6.10	*Thermobispora bispora*	99
7	Stream bank	0.8	Fluvisol	5.90	<1	25	7.11	*Actinomadura rubrobrunea*	98
8	Stream bank	1.3	Fluvisol	6.05	1	15	8.12	*Geobacillus* sp.	77
							8.13	*Paenibacillus caldisaponilyticus*	99
							8.14	*Thermobispora bispora*	96
9	Field	1.3	Luvisol	6.12	<1	4.6	9.15	*Bacillus* sp.	99
							9.16	*Diaphorobacter* sp.	93
							9.17	*Methylobacterium* sp.	95
							9.18	*Thermobispora bispora*	96
10	Grass cover	1.7	Fluvisol	5.40	<1	4.3	10.19	*Anoxybacillus caldiproteolyticus*	99
							10.20	*Geobacillus* sp.	99
11	Field	1.7	Luvisol	6.43	2	12	11.21	*Geobacillus* sp.	99
							11.22	*Geobacillus* sp.	99
							11.23	*Thermobispora bispora*	97
12	Forest	3.5	Luvisol	5.30	<1	3.3	12.24	*Propionibacterium*	100
							12.25	*Microbispora bispora*	99
13	Forest	1.0	Leptosol	5.24	<1	1.3	13.26	*Flectobacillus* sp.	99
							13.27	*Flectobacillus roseus*	99
							13.28	*Geobacillus* sp.	94
							13.29	*Bacillus* sp.	99
							13.30	*Methylobacterium* sp.	97
							13.31	*Microbispora bispora*	96
							13.32	*Actinobacteria*	88
14	Field	1.0	Leptosol	5.45	<1	93	14.33	*Flectobacillus* sp.	99
							14.34	*Flectobacillus roseus*	99
							14.35	*Geobacillus* sp.	94
								*Thermobispora bispora*	99
15	Field	1.2	Cambisol	5.80	<1	8.8	15.36	*Thermobispora bispora*	99
							15.37	*Thermobispora bispora*	99
16	Stream bank	1.5	Fluvisol	6.27	<1	<1	-	*-*	-
17	Field	2.0	Cambisol	5.97	<1	<1	-	*-*	-
18	Field	1.2	Cambisol	6.4	<1	8.7	18.38	*Paenibacillus* sp.	93
							18.39	*Thermobacillus* sp.	95
							18.40	*Aeribacillus* sp.	99
							18.41	*Blastochloris* sp.	87
							18.42	*Thermobispora bispora*	99
19	Field	2.3	Fluvisol	6.42	<1	<1	-	*-*	-
20	Grass cover	1.2	Fluvisol	5.93	<1	7.7	20.43	*Geobacillus* sp.	99
							20.44	*Thermobispora bispora*	99
							20.45	*Thermobispora bispora*	99
21	Field	1.2	Fluvisol	5.30	<1	92	21.46	*Geobacillus debilis*	99
							21.47	*Thermobispora bispora*	99
22	Stream bank	3.5	Fluvisol	6.25	<1	15	22.48	*Paenibacillus* sp.	88
							22.49	*Alicyclobacillus*	90
23	Stream bank	3.5	Fluvisol	5.57	1	140	23.50	*Geobacillus caldoxylosilyticus*	99
								*Thermobispora bispora*	99
							23.51		
24	Orchard	3.5	Cambisol	6.10	1	22	24.52	*Aeribacillus* sp.	99
							24.53		
25	Field	2.3	Chernozem	5.75	<1	<1	-	*-*	-
26	Forest	1.3	Cambisol	5.03	<1	32	26.54	*Alicyclobacillus*	97
							26.55	*Thermobacillus* sp.	95
							26.56	*Blastochloris* sp.	96
							26.57	*Actinomadura* sp.	96
27	Grass cover	0.8	Cambisol	6.50	<1	24	27.58	*Thermobispora bispora*	96
							27.59	*Thermobispora bispora*	97
							27.60	*Geobacillus thermoglucosidasius*	96
								*Parageobacillus thermoglucosidans*	
							27.61		99
28	Forest	1.2	Cambisol	5.23	<1	9.6	28.62	*Thermobispora bispora*	99
							28.63	*Kyrpidia tusciae*	96
29	Grass cover	1.2	Chernozem	6.65	<1	89	29.64	*Tuberibacillus sp.*	99
							29.65	*Paennibacillus sp.*	91
							29.66	*Paenibacillus sp.*	89
							29.67	*Paenibacillus sp.*	94
							29.68	*Anoxybacillus caldiproteolyticus*	99
								*Anoxybacillus caldiproteolyticus*	
							29.69		99
30	Grass cover	1.7	Chernozem	6.77	<1	18	30.70	*Thermobispora bispora*	99
							30.71	*Thermobispora bispora Geobacillus* sp.	99
							30.72	*Bacillus sp.*	99
							30.73		80
31	Field	3.0	Fluvisol	5.81	<1	<1	-	*-*	-
32	Grass cover	2.0	Luvisol	6.12	<1	<1	-	*-*	-
33	Grass cover	2.0	Luvisol	6.14	<1	<1	-	*-*	-
34	Field	1.4	Luvisol	6.72	<1	70	34.74	*Dokdonella* sp.	97
							34.75	*Thermobispora bispora*	99
							34.76	*Thermobispora bispora*	99
35	Field	1.4	Luvisol	5.90	<1	140	35.77	*Paenibacillus ehimensis*	93
							35.78	*Thermoanaerobacterium*	87
36	Field	1.0	Leptosol	6.10	1	5.1	6.79	*Caldicellulosiruptor* sp.	97
							36.80	*Pelomonas aquatica*	99
							36.81	*Methylobacterium* sp.	98
							36.82	*Propionibacterium* sp.	99
37	Stream bank	1.0	Cambisol	6.19	1	16	37.83	*Comamonadaceae bacterium*	99
							37.84	*Thermobispora bispora*	99
38	Stream bank	1.2	Chernozem	6.61	<1	31	38.85	*Geobacillus* sp.	98
							38.86	*Thermobispora bispora*	98
39	Field	1.7	Fluvisol	6.7	2	31	39.87	*Aeribacillus* sp.	99
							39.88	*Geobacillus* sp.	100
							39.89	*Thermobispora bispora*	96
40	Forest	3.0	Fluvisol	5.59	<1	<1	-	*-*	-
41	Field	1.7	Fluvisol	6.75	2	13	41.90	*Paenibacillus sp.*	95
							41.91	*Thermobispora bispora*	98

^a^ organic carbon content in the soil; ^b^ number of thermophilic degrader CFU counted in native soil before the enrichment procedure; ^c^ number of thermophilic degrader CFU counted after the enrichment procedure; ^d^ percentage of identity of the isolated sequence in comparison with the database entry.

## Abbreviations

PBAT	Poly(butylene adipate-co-terephthalate)
NGS	Next generation sequencing
PCR-DGGE	Polymerase chain reaction-denaturing gradient gel electrophoresis
TPS	Thermoplastic starch
PLA	Poly(lactic acid)
TPSu	Thermoplastic starch with urea as plasticizer
PVA	Poly(vinyl) alcohol
SEM	Scanning electron microscopy
CFU	Colony forming units

## References

[1] Liu J., Xu Y., Xia T., Xue C., Liu L., Chang P., Wang D., Sun X. (2020). Oxidized Oligosaccharides Stabilize Rehydrated Sea Cucumbers against High-Temperature Impact. Int. J. Mol. Sci..

[2] Wang H., Qian J., Ding F. (2018). Emerging Chitosan-Based Films for Food Packaging Applications. J. Agric. Food Chem..

[3] Lubkowski K., Smorowska A., Grzmil B., Kozłowska A. (2015). Controlled-Release Fertilizer Prepared Using a Biodegradable Aliphatic Copolyester of Poly(butylene succinate) and Dimerized Fatty Acid. J. Agric. Food Chem..

[4] Sintim H.Y., Bary A.I., Hayes D.G., Wadsworth L.C., Anunciado M.B., English M.E., Bandopadhyay S., Schaeffer S.M., Debruyn J.M., Miles C.A. (2020). In situ degradation of biodegradable plastic mulch films in compost and agricultural soils. Sci. Total Environ..

[5] Sartore L., Schettini E., De Palma L., Brunetti G., Cocozza C., Vox G. (2018). Effect of hydrolyzed protein-based mulching coatings on the soil properties and productivity in a tunnel greenhouse crop system. Sci. Total Environ..

[6] Weng Y.-X., Jin Y., Meng Q.-Y., Wang L., Zhang M., Wang Y. (2013). Biodegradation behavior of poly(butylene adipate-co-terephthalate) (PBAT), poly(lactic acid) (PLA), and their blend under soil conditions. Polym. Test..

[7] Wang J., Wang H., Chen E., Chen Y., Wu T. (2020). Enhanced Photodegradation Stability in Layered Zinc Phenylphosphonate. Polymers.

[8] Pietrosanto A., Scarfato P., Di Maio L., Nobile M.R., Incarnato L. (2020). Evaluation of the Suitability of Poly(Lactide)/Poly(Butylene-Adipate-co-Terephthalate) Blown Films for Chilled and Frozen Food Packaging Applications. Polymers.

[9] Witt U., Müller R.-J., Deckwer W.-D. (1997). Biodegradation behavior and material properties of aliphatic/aromatic polyesters of commercial importance. J. Environ. Polym. Degrad..

[10] Müller R.-J., Kleeberg I., Deckwer W.-D. (2001). Biodegradation of polyesters containing aromatic constituents. J. Biotechnol..

[11] Muroi F., Tachibana Y., Kobayashi Y., Sakurai T., Kasuya K.-I. (2016). Influences of poly(butylene adipate-co-terephthalate) on soil microbiota and plant growth. Polym. Degrad. Stab..

[12] Sangroniz A., González A., Martin L., Irusta L., Iriarte M., Etxeberria A. (2018). Miscibility and degradation of polymer blends based on biodegradable poly(butylene adipate-co-terephthalate). Polym. Degrad. Stab..

[13] Morro A., Catalina F., Sánchez-León E., Abrusci C. (2018). Photodegradation and Biodegradation under Thermophile Conditions of Mulching Films Based on Poly(Butylene Adipate-co-Terephthalate) and Its Blend with Poly(Lactic Acid). J. Polym. Environ..

[14] Stloukal P., Verney V., Commereuc S., Rychly J., Matisova-Rychlá L., Pis V., Koutny M. (2012). Assessment of the interrelation between photooxidation and biodegradation of selected polyesters after artificial weathering. Chemosphere.

[15] Kijchavengkul T., Auras R., Rubino M., Ngouajio M., Fernandez R.T. (2008). Assessment of aliphatic–aromatic copolyester biodegradable mulch films. Part II: Laboratory simulated conditions. Chemosphere.

[16] Ahmed T., Shahid M., Azeem F., Rasul I., Shah A.A., Noman M., Hameed A., Manzoor N., Manzoor I., Muhammad S. (2018). Biodegradation of plastics: Current scenario and future prospects for environmental safety. Environ. Sci. Pollut. Res..

[17] Kleeberg I., Welzel K., Vandenheuvel J., Müller R.-J., Deckwer W.-D. (2005). Characterization of a New Extracellular Hydrolase fromThermobifida fuscaDegrading Aliphatic−Aromatic Copolyesters. Biomacromolecules.

[18] Witt U., Einig T., Yamamoto M., Kleeberg I., Deckwer W.-D., Müller R.-J. (2001). Biodegradation of aliphatic–aromatic copolyesters: Evaluation of the final biodegradability and ecotoxicological impact of degradation intermediates. Chemosphere.

[19] Perz V., Bleymaier K., Sinkel C., Kueper U., Bonnekessel M., Ribitsch D., Guebitz G.M. (2016). Substrate specificities of cutinases on aliphatic–aromatic polyesters and on their model substrates. New Biotechnol..

[20] Kawai F., Oda M., Tamashiro T., Waku T., Tanaka N., Yamamoto M., Mizushima H., Miyakawa T., Tanokura M. (2014). A novel Ca2+-activated, thermostabilized polyesterase capable of hydrolyzing polyethylene terephthalate from Saccharomonospora viridis AHK190. Appl. Microbiol. Biotechnol..

[21] Thumarat U., Nakamura R., Kawabata T., Suzuki H., Kawai F. (2011). Biochemical and genetic analysis of a cutinase-type polyesterase from a thermophilic Thermobifida alba AHK119. Appl. Microbiol. Biotechnol..

[22] Kleeberg I., Hetz C., Kroppenstedt R.M., Müller R.-J., Deckwer W.-D. (1998). Biodegradation of Aliphatic-Aromatic Copolyesters by Thermomonospora fusca and Other Thermophilic Compost Isolates. Appl. Environ. Microbiol..

[23] Muroi F., Tachibana Y., Soulenthone P., Yamamoto K., Mizuno T., Sakurai T., Kobayashi Y., Kasuya K.-I. (2017). Characterization of a poly(butylene adipate- co -terephthalate) hydrolase from the aerobic mesophilic bacterium Bacillus pumilus. Polym. Degrad. Stab..

[24] Tan F.T., Cooper D.G., Marić M., Nicell J.A. (2008). Biodegradation of a synthetic co-polyester by aerobic mesophilic microorganisms. Polym. Degrad. Stab..

[25] Kasuya K.-I., Ishii N., Inoue Y., Yazawa K., Tagaya T., Yotsumoto T., Kazahaya J.-I., Nagai D. (2009). Characterization of a mesophilic aliphatic–aromatic copolyester-degrading fungus. Polym. Degrad. Stab..

[26] Boonmee J., Kositanont C., Leejarkpai T. (2016). Biodegradation of poly(Lactic acid), poly(hydroxybutyrate-co-hydroxyvalerate), poly(butylene succinate) and poly(butylene adipate-co-terephthalate) under anaerobic and oxygen limited thermophilic conditions. Environ. Asia.

[27] Witt U., Müller R.-J., Deckwer W.-D. (1995). New biodegradable polyester-copolymers from commodity chemicals with favorable use properties. J. Environ. Polym. Degrad..

[28] Shah A.A., Eguchi T., Mayumi D., Kato S., Shintani N., Kamini N.R., Nakajima-Kambe T. (2013). Purification and properties of novel aliphatic-aromatic co-polyesters degrading enzymes from newly isolated Roseateles depolymerans strain TB-87. Polym. Degrad. Stab..

[29] Perz V., Hromic A., Baumschlager A., Steinkellner G., Pavkov-Keller T., Gruber K., Bleymaier K., Zitzenbacher S., Zankel A., Mayrhofer C. (2016). An Esterase from Anaerobic Clostridium hathewayi Can Hydrolyze Aliphatic–Aromatic Polyesters. Environ. Sci. Technol..

[30] Saadi Z., Cesar G., Bewa H., Benguigui L. (2013). Fungal Degradation of Poly(Butylene Adipate-Co-Terephthalate) in Soil and in Compost. J. Polym. Environ..

[31] Marten E., Müller R.-J., Deckwer W.-D. (2005). Studies on the enzymatic hydrolysis of polyesters. II. Aliphatic–aromatic copolyesters. Polym. Degrad. Stab..

[32] Šerá J., Stloukal P., Jančová P., Verney V., Pekařová S., Koutny M. (2016). Accelerated Biodegradation of Agriculture Film Based on Aromatic–Aliphatic Copolyester in Soil under Mesophilic Conditions. J. Agric. Food Chem..

[33] Seligra P.G., Moura L.E., Famá L., Druzian J.I., Goyanes S. (2016). Influence of incorporation of starch nanoparticles in PBAT/TPS composite films. Polym. Int..

[34] Asante P., Armstrong G.W., Adamowicz W.L. (2011). Carbon sequestration and the optimal forest harvest decision: A dynamic programming approach considering biomass and dead organic matter. J. For. Econ..

[35] Neilson J.W., Jordan F.L., Maier R.M. (2013). Analysis of artifacts suggests DGGE should not be used for quantitative diversity analysis. J. Microbiol. Methods.

[36] Waterhouse A.M., Procter J.B., Martin D.M.A., Clamp M., Barton G.J. (2009). Jalview Version 2—A multiple sequence alignment editor and analysis workbench. Bioinformatics.

[37] Jones R.J.A., Houšková B., Bullock P., Montanarella L. (2005). Soil Resources of Europe.

[38] Banerjee S., Baah-Acheamfour M., Carlyle C.N., Bissett A., Richardson A.E., Siddique T., Bork E.W., Chang S.X. (2015). Determinants of bacterial communities in Canadian agroforestry systems. Environ. Microbiol..

[39] Tawakaltu A.-A., Egwim E.C., Ochigbo S.S., Ossai P.C. (2015). Effect of Acetic Acid and Citric Acid Modification on Biodegradability of Cassava starch Nanocomposite Films. J. Mater. Sci. Eng. B.

[40] Ivanič F., Kováčová M., Chodák I. (2019). The effect of plasticizer selection on properties of blends poly(butylene adipate-co-terephthalate) with thermoplastic starch. Eur. Polym. J..

[41] Uchida H., Nakajima-Kambe T., Shigeno-Akutsu Y., Nakahara T., Nomura N., Tokiwa Y. (2000). Properties of a bacterium which degrades solid poly(tetramethylene succinate)-co-adipate, a biodegradable plastic. FEMS Microbiol. Lett..

[42] Weisburg W.G., Barns S.M., A Pelletier D., Lane D.J. (1991). 16S ribosomal DNA amplification for phylogenetic study. J. Bacteriol..

[43] Muyzer G., De Waal E.C., Uitterlinden A.G. (1993). Profiling of complex microbial populations by denaturing gradient gel electrophoresis analysis of polymerase chain reaction-amplified genes coding for 16S rRNA. Appl. Environ. Microbiol..

[44] Husárová L., Pekařová S., Stloukal P., Kucharzcyk P., Verney V., Commereuc S., Ramone A., Koutny M. (2014). Identification of important abiotic and biotic factors in the biodegradation of poly(l-lactic acid). Int. J. Biol. Macromol..

[45] Das M., Royer T.V., Leff L.G. (2006). Diversity of Fungi, Bacteria, and Actinomycetes on Leaves Decomposing in a Stream. Appl. Environ. Microbiol..

[46] Větrovský T., Baldrian P., Morais D. (2018). SEED 2: A user-friendly platform for amplicon high-throughput sequencing data analyses. Bioinformatics.

[47] McMurdie P.J., Holmes S. (2013). phyloseq: An R Package for Reproducible Interactive Analysis and Graphics of Microbiome Census Data. PLoS ONE.

[48] Quast C., Pruesse E., Yilmaz P., Gerken J., Schweer T., Yarza P., Peplies J., Glöckner F.O. (2012). The SILVA ribosomal RNA gene database project: Improved data processing and web-based tools. Nucleic Acids Res..

